# Extracorporeal life support devices and strategies for management of acute cardiorespiratory failure in adult patients: a comprehensive review

**DOI:** 10.1186/cc13865

**Published:** 2014-05-09

**Authors:** Kiran Shekar, Daniel V Mullany, Bruce Thomson, Marc Ziegenfuss, David G Platts, John F Fraser

**Affiliations:** 1 Critical Care Research Group, Adult Intensive Care Services, The Prince Charles Hospital and The University of Queensland, Rode Road, Chermside, Brisbane, Queensland 4032, Australia; 2 Department of Cardiothoracic Surgery, The Prince Charles Hospital, Rode Road, Chermside, Brisbane, Queensland 4032, Australia; 3 Queensland Advanced Heart Failure and Cardiac Transplant Unit, The Prince Charles Hospital, Rode Road, Chermside, Brisbane, Queensland 4032, Australia

## Abstract

Evolution of extracorporeal life support (ECLS) technology has added a new dimension to the intensive care management of acute cardiac and/or respiratory failure in adult patients who fail conventional treatment. ECLS also complements cardiac surgical and cardiology procedures, implantation of long-term mechanical cardiac assist devices, heart and lung transplantation and cardiopulmonary resuscitation. Available ECLS therapies provide a range of options to the multidisciplinary teams who are involved in the time-critical care of these complex patients. While venovenous extracorporeal membrane oxygenation (ECMO) can provide complete respiratory support, extracorporeal carbon dioxide removal facilitates protective lung ventilation and provides only partial respiratory support. Mechanical circulatory support with venoarterial (VA) ECMO employed in a traditional central/peripheral fashion or in a temporary ventricular assist device configuration may stabilise patients with decompensated cardiac failure who have evidence of end-organ dysfunction, allowing time for recovery, decision-making, and bridging to implantation of a long-term mechanical circulatory support device and occasionally heart transplantation. In highly selected patients with combined severe cardiac and respiratory failure, advanced ECLS can be provided with central VA ECMO, peripheral VA ECMO with timely transition to venovenous ECMO or VA-venous ECMO upon myocardial recovery to avoid upper body hypoxia or by addition of an oxygenator to the temporary ventricular assist device circuit. This article summarises the available ECLS options and provides insights into the principles and practice of these techniques. One should emphasise that, as is common with many emerging therapies, their optimal use is currently not backed by quality evidence. This deficiency needs to be addressed to ensure that the full potential of ECLS can be achieved.

## Review

### Introduction

Extracorporeal life support (ECLS) is a therapeutic option increasingly used in the management of patients with cardiorespiratory failure that is refractory to maximal conventional treatment [1,2]. This support may facilitate therapeutic intervention, bridge to recovery, bridge to a long-term support device, heart or lung transplantation, or bridge to palliation. Despite the renewed interest in ECLS technology following the 2009 H1N1 influenza pandemic [3], there is a lack of definitive evidence regarding its routine application. Current ECLS equipment has evolved to allow a plethora of perfusion strategies enabling tailored temporary support for patients and the ability to transition between configurations. A number of factors limit more frequent utilisation. These factors include challenges in patient selection, choice of an appropriate strategy, technical aspects of initiation and maintenance, and minimising complications [4].

This review provides a summary of the available ECLS options and cannulation techniques for short-term support of adult patients with cardiorespiratory failure that is refractory to conventional treatment.

## Extracorporeal respiratory support for respiratory failure

### Extracorporeal membrane oxygenation

Details about the technology, principles and practice of extracorporeal membrane oxygenation (ECMO) can be found elsewhere [5,6]. Venovenous (VV) ECMO is predominantly used as a rescue therapy for selected patients with acute respiratory distress syndrome (ARDS) and refractory hypoxia [1,7,8]. Patients with refractory hypoxia due to ARDS continue to have a high mortality, in the order of 70 to 90%. Whether VV ECMO further improves survival in this group of patients when compared with lung-protective ventilation (LPV) and adjuncts such as neuromuscular blockade [9] and prone ventilation [10] is yet to be established. Recent studies demonstrating harm or no benefit with the use of high-frequency oscillation in patients with moderate to severe ARDS exclude this technique as a routine rescue option for refractory hypoxia and may further expand the scope of VV ECMO [11,12]. Despite unfavourable results in the early studies, the CESAR trial showed improved disability-free survival at 6 months in 90 patients who were randomised to receive ECMO (37% vs. 53% on LPV, *P* = 0.03) [13]. However, 22 of these patients did not receive ECMO and a majority of them improved with LPV. The study was criticised for lack of standardisation of LPV in the control group. This may not be seen as definitive evidence supporting the use of VV ECMO. However, the CESAR trial and the UK data from patients with H1N1-related ARDS do confirm that referral to an ECMO centre may lower hospital mortality compared with matched non-ECMO-referred patients [14].

Analysis of 2009 H1N1 pandemic data [3,15-18] demonstrates that while VV ECMO may be in equipoise with LPV, it may play a vital role in younger patients with critical oxygenation who have fewer failed organ systems and fail LPV [8]. Although VV ECMO is relatively easy to institute technically, the complexities relate more to the availability of the service, practicalities of transfer to an ECLS centre, timing and patient selection, care of the patient on ECMO and minimising complications [4]. Patients often need to be retrieved whilst supported by ECMO, and data suggest that this can be undertaken safely in trained hands with an appropriate system [19]. VV ECMO and the more portable proprietary extracorporeal respiratory support devices such as Cardiohelp™ (Maquet Cardiopulmonary AG, Hirrlingen, Germany) can be utilised for transport [20].

A number of configurations of VV ECMO can be applied based on individual patient requirements (Table 1). Although there are many other factors, patient arterial oxygenation is critically dependent on the fraction of total cardiac output passing through the oxygenator while adequate carbon dioxide (CO_2_) clearance can still occur with lower blood flows [21]. The bicaval dual-lumen Avalon™ cannula (Avalon Laboratory, Los Angeles, CA, USA) inserted through the internal jugular vein allows single-site cannulation for VV ECMO [22], but flows are unlikely to be as high compared with the use of two large venous drainage cannulae positioned in the venae cavae via internal jugular and femoral veins. Additionally, meticulous positioning is required, usually with transoesophageal echocardiography. Use of a dual-lumen cannula in the neck may facilitate mobilisation in bed, extubation and rehabilitation in patients who receive prolonged ECMO support [22].

**Table 1:** Available extracorporeal respiratory support devices and strategies

**ECLS strategy**	**Principle indication(s)**
VV ECMO standard (femoral vein–femoral vein)	Default strategy for complete extracorporeal respiratory support
VV ECMO (dual-lumen cannula)	Complete or partial respiratory support predominantly
	Bridge to lung transplant
VV ECMO high flow (SVC and IVC access)	Complete respiratory support for larger patients; for example, male weight >90 kg
VV ECMO high flow with two oxygenators in parallel	Complete respiratory support for very large patients; for example, male weight >120 kg
Femoral VV with pump (iLA™Activve; Novalung GmbH, Hechingen, Germany)	Complete or partial respiratory support
Pulmonary artery–left atrium pumpless with oxygenator (iLA™; Novalung GmbH)	Bridge to lung transplant
	Salvage for refractory hypoxia during complete respiratory support on VV ECMO
	Salvage for severe pulmonary hypertension with normal left heart
Femoral arterio-venous pumpless (iLA™; Novalung GmbH)	Partial respiratory support only in a very haemodynamically stable patient
VV ECCOR (Hemolung™; Alung Technologies, Pittsburgh, PA, USA)	Partial respiratory support

Complete respiratory support, oxygenation and carbon dioxide removal; partial respiratory support, carbon dioxide removal and some or no oxygenation. ECCOR, extracorporeal carbon dioxide removal; ECLS, extracorporeal life support; ECMO, extracorporeal membrane oxygenation; IVC, inferior vena cava, iLA, interventional lung assist; SVC, superior vena cava; VV, venovenous.

### Extracorporeal carbon dioxide removal

Hypercapnia and respiratory acidosis, although usually well tolerated, is a barrier to implementing ultra-protective ventilation [21]. This barrier has renewed interest in extracorporeal technologies that facilitate extracorporeal CO_2_ removal (ECCOR). Refinements in technology [21,23] have resulted in fewer complications when used as adjuncts to LPV [24], but definitive evidence is lacking [25].

ECCOR requires an arterial or venous access cannula, a pump to drain blood during venous access, a membrane lung and a return venous cannula. Heparin-coated wire-reinforced cannulae may be placed percutaneously in a femoral–femoral or a femoral–jugular orientation. Alternatively, a wire-reinforced double-lumen catheter may be inserted under ultrasound guidance via the right internal jugular vein with the drainage port positioned in the intra-hepatic inferior vena cava and the return port in the right atrium [21,26]. A flow of fresh gas containing little or no CO_2_ is utilised to create a diffusion gradient across the membrane and allows CO_2_ removal. While ECMO necessitates high blood flow rates (5 to 7 l/minute) to ensure optimal oxygenation, ECCOR allows CO_2_ removal at much lower blood flow rates (<1 l/minute) due to significant differences in CO_2_ and oxygen kinetics [21,23]. Although lower blood flows can be achieved with smaller cannulae with greater ease, vascular complications may still occur especially with arterial cannulation.

**Figure 1:**
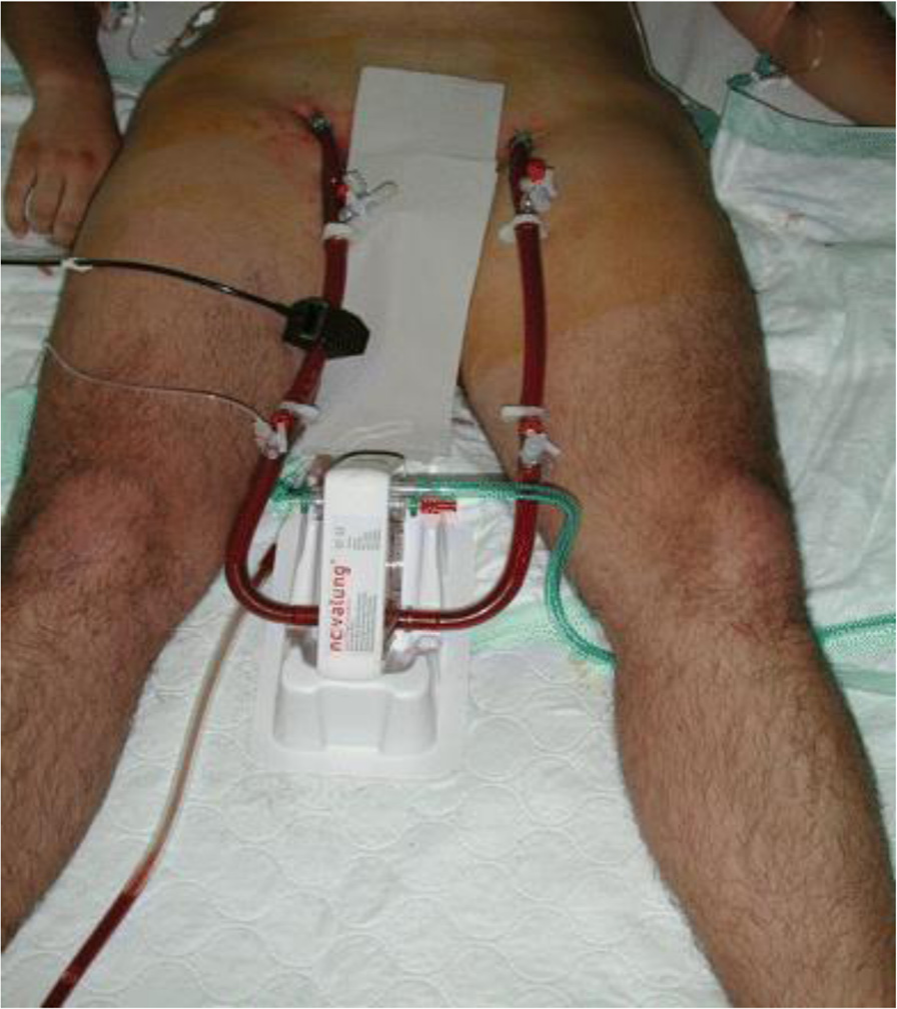
**Interventional lung assist device (iLA™; NovaLung GmbH, Talheim, Germany) for pumpless arterio-venous carbon dioxide removal.** Reproduced with permission from [27].

Various novel ECCOR devices are currently available to facilitate LPV and are reviewed in detail elsewhere [21,23]. The available and emerging devices are summarised in Table 1. The pumpless interventional lung assist iLA™ membrane ventilator marketed by Novalung GmbH (Hechingen, Germany) is a low-gradient device (Figure 1) that can be employed peripherally (femoral artery access and femoral vein return) and allows complete CO_2_ removal in patients with adequate oxygenation and robust haemodynamics. There have been reports of its successful use in patients with ARDS [28] and severe asthma [29] and as a bridge to lung transplantation [30]. However, the risks of arterial access have to be carefully considered in these patients. The pulmonary artery–left atrial configuration of the same device has been used as a bridge to lung transplantation particularly in those who have significant pulmonary hypertension [31]. A VV configuration of the membrane oxygenator with a pump (iLA™ Activve; Novalung GmbH) is also available for partial or complete respiratory support [32].

Devices such as the Decap™ system (Hemodec, Salerno, Italy) that serve the dual purpose of renal replacement therapy and ECCOR are also available [33]. By combining the membrane lung and the pump into a single unit, the Hemolung™ (Alung Technologies, Pittsburgh, PA, USA) achieves efficient CO_2_ removal at flows between 400 and 600 ml/minute [21] using dual-lumen catheters similar to those used for renal replacement therapy. These low-flow systems provide partial CO_2_ removal only and do not provide any oxygenation benefit. Even though modern low-flow VV ECCOR devices reportedly require a lower degree of anticoagulation, concerns remain over risks of circuit thrombosis. Other emerging technologies such as intravascular gas exchange and respiratory dialysis [21] are beyond the scope of this article. Similarly, bridging the acutely ill patients to lung transplantation [34,35] with ECLS is a highly specialised service beyond the scope of this review.

Extracorporeal respiratory support can thus be provided with ECCOR or ECMO depending on the lung pathology, pulmonary compliance and oxygenation and decarboxylation requirements of an individual patient. ECMO and ECCOR can also bridge highly selected patients to lung transplantation.

## Extracorporeal life support in acute cardiac failure

Providing temporary mechanical circulatory support (MCS) support to patients with acute refractory cardiac failure using ECLS techniques is a rapidly evolving area; intervention may be time critical and mortality is higher than ECLS for isolated respiratory failure [36,37]. The use of ECLS in the setting of cardiopulmonary resuscitation is discussed elsewhere [38,39]. Patient outcomes with the use of long-term ventricular assist devices (VADs) in cardiogenic shock (INTERMACS class 1) are poor [40,41]. Recently published International Society for Heart and Lung Transplantation Guidelines for MCS provide recommendations for long-term MCS options for patients with cardiac failure [42], and these are not discussed in this article. These guidelines strongly recommend consideration of the use of temporary MCS in patients with multiorgan failure, with sepsis or on mechanical ventilation to allow successful optimisation of their clinical status and neurologic assessment prior to placement of a long-term MCS device [42].

The severity of noncardiac organ system failures can be defined using scoring systems such as the Sequential Organ Failure Assessment score. Severe multiorgan failure (for example, Sequential Organ Failure Assessment score >15) has been considered a contraindication to VV ECMO [43] and similar criteria may be applicable for venoarterial (VA) ECMO or for the use of an ECMO circuit as a temporary VAD. Factors considered in the initial cannulation strategy include: the underlying cause of cardiac dysfunction and projected time course of recovery; the severity of pulmonary dysfunction and projected time course of recovery; the functional reserve of each ventricle; the presence and severity of valvular pathology; risk of arterial access and size of vessels; the severity of coagulopathy and risk of sternotomy; and planned future surgery, such as long-term VAD or transplant.

**Figure 2:**
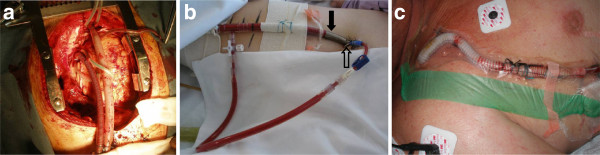
**Venoarterial extracorporeal membrane oxygenation.** Venoarterial extracorporeal membrane oxygenation can be instituted: **(a)** centrally by cannulating the right atrium/inferior venacava and the aorta; **(b)** peripherally using the femoral vein and femoral artery (solid arrow, arterial return cannula; hollow arrow, back-flow cannula for distal limb perfusion); or **(c)** peripherally using the axillary/subclavian artery. The choice is often guided by the clinical setting, the expected duration of support and pulmonary function.

For patients with predominant cardiac failure with preserved pulmonary function, the available MCS devices provide several options (Table 2). Central VA ECMO has been traditionally applied as a bridge to recovery in patients who fail to wean from cardiopulmonary bypass after cardiac surgery (Figure 2). Central VA ECMO outside this setting in adults is uncommon. Femoral VA ECMO (Figure 2) is more commonly used in adults requiring urgent cardiac support because it can be achieved rapidly and a sternotomy is avoided. One of the major limitations of peripheral femoro-femoral VA ECMO is left ventricular (LV) afterload mismatch and inadequate LV decompression/venting. This limitation is particularly so in patients with very low native cardiac output states and severe mitral valve regurgitation, and may result in severe hydrostatic pulmonary oedema in some patients. Although some centres use an intra-aortic balloon pump in conjunction with peripheral VA ECMO to reduce LV afterload and pulmonary congestion, no definitive data exist to support routine use.

**Table 2:** Extracorporeal life support strategies for mechanical circulatory support in isolated cardiac failure

**ECLS strategy**	**Principle indication(s)**
VA ECMO (return femoral artery)	Default strategy for potentially reversible cardiogenic shock of any cause
Central VA ECMO (return aorta)	Failure to wean from cardiopulmonary bypass where recovery expected within 7 days
	Salvage for small patients with cardiogenic shock where femoral arterial access inadequate
VA ECMO (return axillary artery)	Reversible cardiogenic shock where high flows not required
	Reversible cardiogenic shock with lower-limb vascular disease
Centrimag™ (Levitronix LLC, Waltham, MA, USA) LVAD (access left atrium/left ventricle, return aorta)	Isolated LV support where recovery is expected in 8 weeks
Centrimag™ (Levitronix LLC) RVAD (access right atrium, return pulmonary artery)	Isolated RV support where recovery is expected in 8 weeks
Centrimag™ (Levitronix LLC) BiVAD	Biventricular support where recovery is expected in 8 weeks
TandemHeart (CardiacAssist, Inc., Pittsburgh, PA, USA) percutaneous LVAD (access left atrium via femoral vein, return femoral artery	Isolated LV support
Impella™ (Abiomed, Aachen, Germany) percutaneous LVAD (access femoral artery)	Isolated LV support
Peripheral VA ECMO + Impella™ (Abiomed) percutaneous LVAD	Isolated LV support with better LV decompression
Implantable LVAD + temporary RVAD (±oxygenator)	Met criteria for LVAD but unexpected reversible RV dysfunction occurred

BiVAD, biventricular assist device; ECLS, extracorporeal life support; ECMO, extracorporeal membrane oxygenation; LV, left ventricular; LVAD, left ventricular assist device; RV, right ventricular; RVAD, right ventricular assist device; VA, venoarterial.

LV and aortic root stasis from lack of cardiac ejection and failure of aortic valve opening may result in catastrophic intracardiac and aortic root thrombosis. Increased anticoagulation to minimise this risk may heighten the risk of significant bleeding. Minimally invasive strategies such as percutaneous transseptal left atrial decompression [44] and subxiphoid surgical approaches to drain the left ventricle [45] have been described to reduce LV distension. The residual atrial defect may require correction once the patient has been weaned from mechanical support. Use of a percutaneously inserted VAD (Impella™; Abiomed, Aachen, Germany) to decompress the left ventricle has also been reported in this setting [46], alleviating the need for a high-risk septostomy or surgical venting.

Femoral VA ECMO is also limited by femoral arterial size, and thus cannula size and the requirement for distal limb perfusion. Given its less invasive nature (compared with thoracic access), peripheral VA ECMO – with attention to optimal LV afterload, minimising LV distension with optimal fluid and inotrope therapy, anticoagulation and pulmonary management – is a viable first-line option for patients with isolated acute cardiac failure refractory to conventional management.

**Figure 3:**
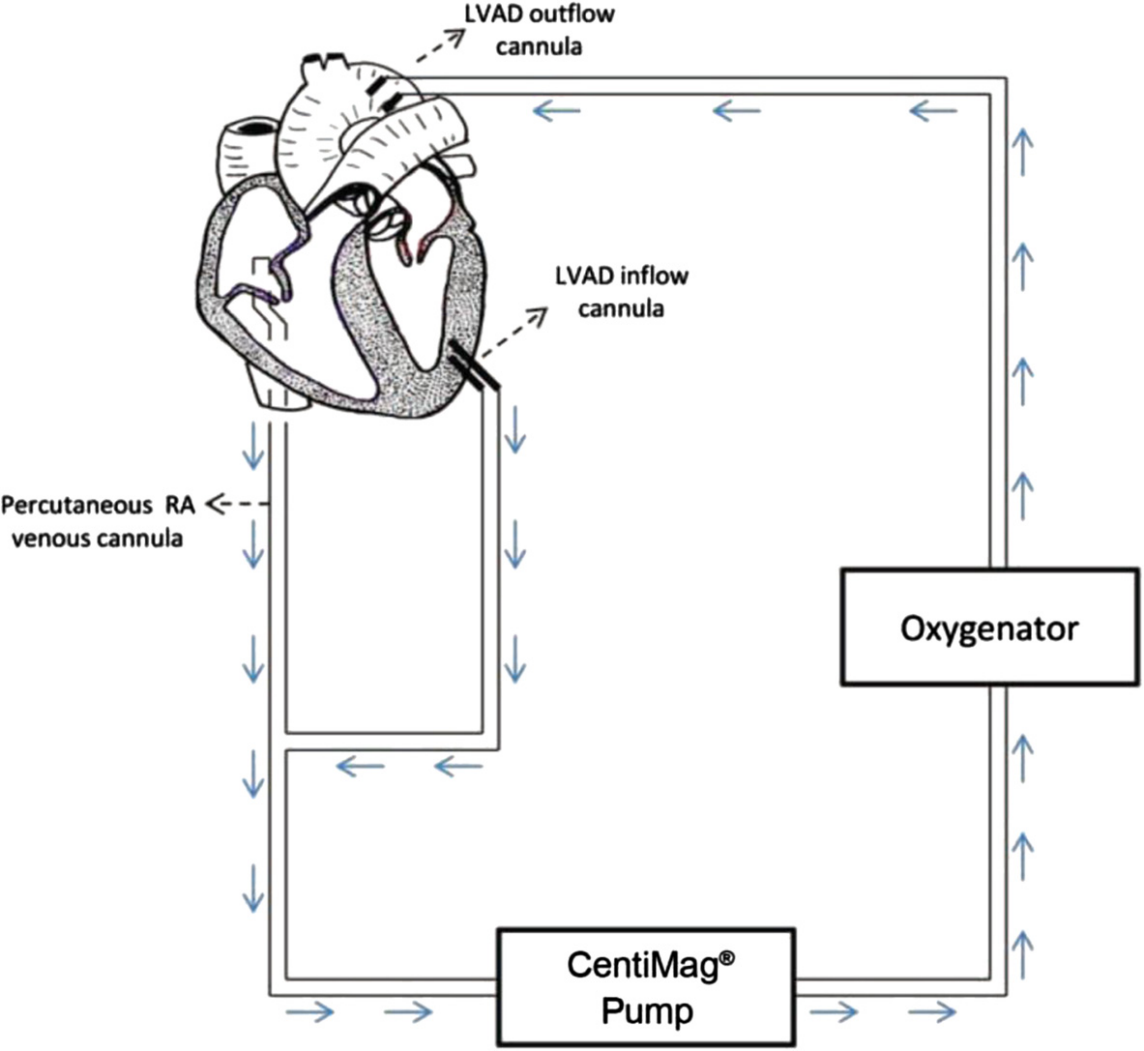
**Biventricular assist device along with respiratory support provided by the oxygenator in the circuit.** The hybrid Centrimag™ (Levitronix LLC, Waltham, MA, USA) extracorporeal membrane oxygenation system can be used as a biventricular assist device along with respiratory support provided by the oxygenator in the circuit. Reproduced with permission from [47]. LVAD, left ventricular assist device; RA, right atrium.

**Figure 4:**
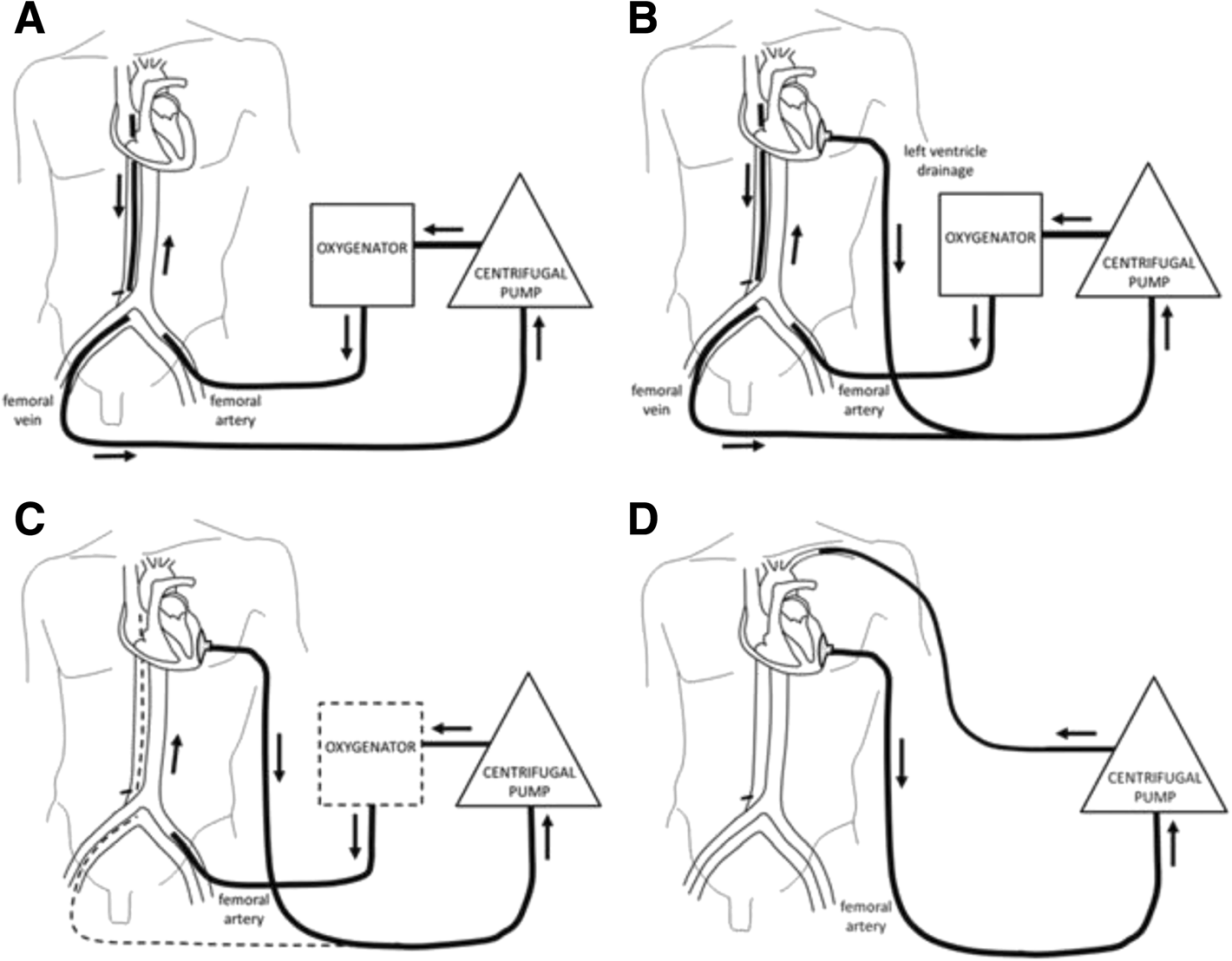
**From venoarterial extracorporeal membrane oxygenation (ECMO) to use of ECMO as a temporary ventricular assist device. (A)** Emergent femoro-femoral venoarterial extracorporeal membrane oxygenation. **(B)** Left ventricular apical cannulation and decompression. **(C)** Right ventricular recovery and isolated temporary left ventricular support. **(D)** Axillary artery cannulation to facilitate mobilisation. Reproduced with permission from [48].

The limitations of peripheral VA ECMO have prompted the use of ECMO devices [47] to facilitate ventricular unloading by changing to a temporary left ventricular assist device (LVAD) or a biventricular assist device configuration (Figure 3). Any perfusion strategy that creates a right to left shunt requires an oxygenator in the circuit. Oxygenators may additionally provide temperature control. This strategy effectively provides biventricular support and gas exchange through a single pump configuration with the ability to cease right ventricular (RV) support when not required. However, this configuration requires sternotomy and cannulation of the left ventricle (or left atrium) and aorta. A reoperation (sternotomy or thoracotomy) is then required for explantation of the cannulae upon cardiac recovery or for implantation of a long-term mechanical assist device. Less invasive techniques for temporary cardiorespiratory support including a transition strategy to an intermediate-term support configuration [48] allowing mobilisation have been described (Figure 4). Although this configuration requires a left thoracotomy, sternotomy is avoided, potentially reducing risk for subsequent surgery in the absence of cardiac recovery (long-term VAD implantation as a bridge to destination or heart transplantation).

Temporary RV support can be provided with the Centrimag™ ECMO system (Levitronix LLC, Waltham, MA, USA) through percutaneous femoral venous access to the right atrium and return to the pulmonary artery via a cannulated exteriorised Dacron graft. This strategy is described for temporary support of the RV with insertion of a long-term LVAD but is applicable to other causes of severe isolated RV dysfunction. An oxygenator can be included in the circuit to ensure adequate oxygenation, CO_2_ removal and temperature regulation. Upon RV recovery, the graft can be ligated and buried upon decannulation without re-sternotomy.

Percutaneously inserted LVADs such as TandemHeart™ (CardiacAssist, Inc., Pittsburgh, PA, USA) and Impella™ (Abiomed) [49] are potential options for MCS in the acute setting. However, there is a paucity of supportive evidence for their use and the complications with arterial access such as bleeding and limb ischemia cannot be understated. TandemHeart™ utilises a centrifugal pump to drain the left atrial blood from a catheter placed transeptally via the femoral vein and returns it to the femoral artery. The Impella™ device uses an axial pump that is inserted retrogradely across the aortic valve via the femoral artery. These devices provide LV support and lack the ability to provide extracorporeal respiratory support if required. However, there are case reports pertaining to their successful use as RV assist devices and/or biventricular assist devices [50,51].

Even though the third-generation, implantable LVADs designed for long-term MCS are a significant improvement on earlier devices [52], their use in a deteriorating patient with multiorgan dysfunction is associated with poor outcomes and is not currently recommended.

## Advanced extracorporeal life support in severe cardiorespiratory failure

The number of patients with severe combined cardiac and respiratory failure who fail conventional treatment is very small and ECLS in this group is controversial, being considered either heroic or futile. In the setting of pneumonia or sepsis and severe cardiac dysfunction, where feasible, VV ECMO with inotropic support should be the initial perfusion strategy [53]. Septic myocardial depression may improve with management of the sepsis, improved oxygenation and normalisation of respiratory acidosis [54]. Peripheral femoral VA ECMO (Figure 2) may be considered a rescue option for these patients if myocardial depression is profound [55] or if the diagnosis is uncertain and conditions such as myocarditis are considered likely. Use of this strategy in septic patients with multiple organ failure who may have severe coagulation and hepatic dysfunction may be futile. However, heroic measures can result in good outcomes [56].

Upper body hypoxia can occur if myocardial recovery occurs and lung function remains poor. This may be overcome by transition to VV ECMO if myocardial recovery is satisfactory or with the use of VA–venous ECMO, which allows return of oxygenated blood to both arterial and venous sides of the circulation, thereby minimising the risk of upper-body hypoxia. Although peripheral arterial cannulation for VA–venous ECMO is less invasive and is an attractive option, balancing the oxygenation and perfusion needs of an individual patient by regulating the return of oxygenated blood to the underperfused coronary and cerebral circulation may be challenging, and a central configuration may be preferred in this setting. Returning the oxygenated blood in the ascending aorta by cannulating the axillary [57,58] or subclavian artery cannulation has also been described (Figure 2) in this setting. However, axillary artery side graft cannulation may be complicated by ipsilateral upper-limb hyperperfusion and bleeding from the arterial graft [59].

**Figure 5:**
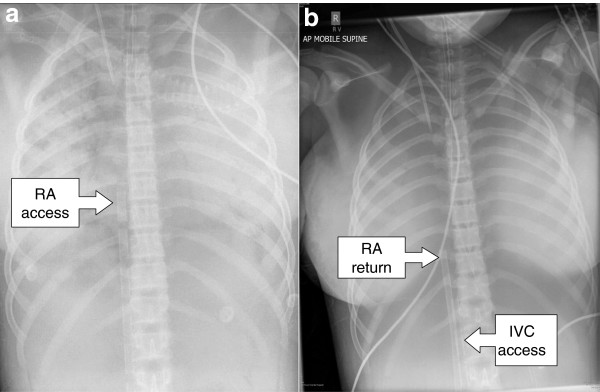
**Patient *in extremis *initially receiving femoro-femoral venoarterial extracorporeal membrane oxygenation (ECMO) with transition to venovenous ECMO.** Patient *in extremis* initially received femoro-femoral venoarterial (VA) ECMO for severe cardiorespiratory failure with transition to venovenous (VV) ECMO on day 4 following satisfactory cardiac recovery. **(a)** Chest X-ray scan shows multistage access cannula in the right atrium (RA) during VA ECMO, **(b)** which was later withdrawn into the inferior vena cava (IVC) during VV ECMO. **(b)** A venous return cannula can also be seen in the right atrium.

A more invasive, high-risk option in this setting includes use of the Centrimag™ ECMO system (Levitronix LLC) as a temporary LVAD/biventricular assist device with an oxygenator in the circuit. The device can be employed in several configurations (Table 3) to support both the left and/or right ventricles and the oxygenator can be removed from the circuit when pulmonary function stabilises. This strategy can support patients for a longer period of time, allowing more time to recover, and minimises the risks of LV distension and thrombosis. This is ideally suited to patients with suspected acute myocarditis in whom myocardial recovery is possible but prolonged support may be required. Alternatively, central VA ECMO may be used in a patient *in extremis*[60,61] when femoral cannulation is expected to be difficult. Regardless of the initial strategy used, transition to VV ECMO (Figure 5) should be considered as soon as adequate cardiac function returns and is pragmatically possible. Continued vigilance as well as prospective risk management of the potential for LV and/or aortic root thrombosis must be considered when exploring specific potential configurations, and must be assessed prior to implantation.

**Table 3:** Advanced extracorporeal life support strategies for cardiac and respiratory support: bridging to intermediate or long-term support may be required

**ECLS strategy**	**Principle indication(s)**
VA ECMO (return femoral artery)	Default strategy for potentially reversible cardiogenic shock of any cause
VA ECMO (return axillary artery)	Reversible cardiogenic shock where high flows are not required
	Reversible cardiogenic shock with lower-limb vascular disease
	Reversible cardiogenic shock with poor gas exchange
VA ECMO (return ascending aorta)	Failure to wean from cardiopulmonary bypass where recovery expected within 7 days
	Salvage for small patients with cardiogenic shock where femoral arterial access inadequate
	Salvage for severe combined cardiac and respiratory failure
VA–venous ECMO	Patients developing circulatory instability on venovenous ECMO
	Salvage for severe combined cardiac and respiratory failure
Venous–pulmonary artery ECMO	Reversible RV dysfunction expected duration up to 2 weeks
Centrimag™ (Levitronix LLC, Waltham, MA, USA) RVAD (femoral access + oxygenator)	Reversible RV dysfunction expected duration up to 2 weeks
Centrimag™ (Levitronix LLC) RVAD (right atrium access + oxygenator)	Reversible isolated RV dysfunction expected duration up to 8 weeks with plan to remove oxygenator and convert to RVAD
Centrimag™ (Levitronix LLC) hybrid (requires oxygenator)	Severe LV after load mismatch on VA ECMO
	Severe combined cardiac and respiratory failure where early RV recovery is expected before intermediate term LV recovery
Implantable LVAD + temporary RVAD (±oxygenator)	Met criteria for LVAD but unexpected reversible RV dysfunction occurred

ECLS, extracorporeal life support; ECMO, extracorporeal membrane oxygenation; LV, left ventricular; LVAD, left ventricular assist device; RV, right ventricular; RVAD, right ventricular assist device; VA, venoarterial.

By providing a range of support options based on the degree of cardiac and respiratory failure (Table 3), ECLS thus redefines the contemporary management of this condition.

## Experimental extracorporeal life support therapies

Several other ECLS cannulation strategies merit consideration and further investigation. These strategies are necessitated by inherent limitations of ECLS therapies such as VV ECMO and VA ECMO and also to make some of the support options less invasive. A summary of these therapies is presented in Table 4.

**Table 4:** Experimental extracorporeal life support options

**ECLS strategy**	**Possible indication(s)**
VV ECMO + atrial septostomy	Refractory hypoxia and/or pulmonary hypertension on VV ECMO avoiding sternotomy
VV ECMO + transeptal return to left atrium	Refractory hypoxia and/or pulmonary hypertension on VV ECMO avoiding sternotomy
Venoarterial ECMO + transeptal access from left atrium and right atrium	Refractory left ventricular distension on venoarterial ECMO

ECLS, extracorporeal life support; ECMO, extracorporeal membrane oxygenation; VV, venovenous.

Refractory hypoxia and severe pulmonary hypertension may be encountered on VV ECMO and is often terminal. Percutaneous transeptal cannulation of the left atrium to return the oxygenated blood has been proposed as an option to avoid a sternotomy, which needs to be further investigated. Similarly, percutaneous transeptal cannulation of the left atrium for access along with percutaneous right atrial cannulation may assist in venting the left ventricle during VA ECMO. Other emerging less invasive ECLS options include hybrid systems that can provide renal replacement therapy and CO_2_ removal.

## Limitations of extracorporeal life support therapies

ECLS therapies are high-risk invasive interventions undertaken in a few specialised centres. Despite the advancements in ECLS technology, the associated complications such as bleeding, thrombosis and infections cannot be underestimated. Apart from the risk profile, the success of ECLS relies heavily on its clinical application. Careful selection of both patients and the ECLS perfusion strategy is the key and not all centres may have the experience or the resources to provide the full complement of ECLS therapies discussed in this paper.

The lack of robust evidence is a significant limitation. The complexities in delivering ECLS include resources and cost-effectiveness, staff training, governance and availability of funding for other programmes such as cardiothoracic surgery, long-term mechanical assist devices, and heart and lung transplantation. Such undertaking may be feasible in resource-rich settings, but significant innovation and refinement is required prior to its widespread use. With minimal improvement in outcomes in ARDS over the years, widespread use of VV ECMO and ECCOR may be a reality in years to come. VA ECMO and its use as a temporary VAD for MCS is a complex undertaking and its use will probably be limited to specialised centres.

## Conclusion

ECLS therapies hold promise and further research is indicated to explore their full potential. Given the small number of patients who receive ECLS for cardiac and/or respiratory support globally, it may not be feasible to generate evidence-based guidelines for all available therapies. However, ongoing refinements in technology, development of minimally invasive techniques, better understanding of the physiological impact of the ECLS circuit (for example, altered pharmacokinetics of vital drugs [62]) and improved clinical delivery may improve patient outcomes. ECLS therapies will probably play a vital future role in the management of adult patients with acute cardiorespiratory failure. Collaboration between global ECLS centres is the key in designing and conducting high-quality clinical trials that will hopefully provide more clarity in patient selection, choice of ECLS device and the appropriate perfusion strategy to be used.

## Abbreviations

ARDS: Acute respiratory distress syndrome; CO2: Carbon dioxide; ECCOR: Extracorporeal carbon dioxide removal; ECLS: Extracorporeal life support; ECMO: Extracorporeal membrane oxygenation; LPV: Lung-protective ventilation; LV: Left ventricular; LVAD: Left ventricular assist device; MCS: Mechanical circulatory support; RV: Right ventricular; VA: Venoarterial; VAD: Ventricular assist device; VV: Venovenous.

## Competing interests

The authors declare that they have no competing interests.
